# *Pf*s230 and *Pf*s48/45 Fusion Proteins Elicit Strong Transmission-Blocking Antibody Responses Against *Plasmodium falciparum*

**DOI:** 10.3389/fimmu.2019.01256

**Published:** 2019-06-05

**Authors:** Susheel K. Singh, Susan Thrane, Bishwanath K. Chourasia, Karina Teelen, Wouter Graumans, Rianne Stoter, Geert-Jan van Gemert, Marga G. van de Vegte-Bolmer, Morten A. Nielsen, Ali Salanti, Adam F. Sander, Robert W. Sauerwein, Matthijs M. Jore, Michael Theisen

**Affiliations:** ^1^Department for Congenital Disorders, Statens Serum Institut, Copenhagen, Denmark; ^2^Centre for Medical Parasitology at Department of International Health, Immunology and Microbiology, University of Copenhagen and Department of Infectious Diseases, Copenhagen University Hospital, Rigshospitalet, Copenhagen, Denmark; ^3^Department of Medical Microbiology, Radboud University Medical Center, Nijmegen, Netherlands

**Keywords:** malaria, vaccines, multivalent, transmission blocking, *Lactococcus lactis*

## Abstract

The *Plasmodium falciparum Pf*s230 and *Pf*s48/45 proteins are expressed during transmission from man to mosquito and are leading candidates for a malaria transmission blocking vaccine. Individually they generate transmission blocking (TB) antibodies in rodent models. Whether the single protein vaccines are suitable to use in field settings will primarily depend on their potency to elicit functional antibodies. We hypothesized that a combination of both proteins will be more potent than each protein individually. Therefore we designed chimeric proteins composed of fragments of both *Pf*s230 and *Pf*s48/45 as well as single protein fragments, and expressed these in *Lactoccus lactis*. Both the individual *Pf*s230 and *Pf*s48/45 fragments and chimeras elicited high levels of functional antibodies in mice. Importantly, one of the chimeric proteins elicited over threefold higher transmission blocking antibody responses than the single antigens alone. Furthermore the immunogenicity of one of the chimeras could be enhanced through coupling to a virus-like particle (VLP). Altogether these data support further clinical development of these novel constructs.

## Introduction

The transmission of *Plasmodium falciparum* from one person to another relies on the generation of male and female gametocytes in the human host that can be picked up and spread by a mosquito. The aim of a malaria transmission blocking vaccine (MTBV) is to effectively block malaria transmission at the population level thereby contributing to malaria elimination.

Several MTBV candidates have been identified by screening monoclonal antibodies generated against *P. falciparum* mosquito stages for TB activity (1–4). Three proteins, *Pf* s48/45, *Pf* s230, and *Pf* s25 are currently targeted as lead candidates for an MTBV. Of these, *Pf* s48/45 and *Pf* s230 are expressed in the gametocyte as it develops from stage III through V in the human host. Shortly, after being taken up by a blood-feeding mosquito, the parasite emerges from the RBC as a gamete and after a few rounds of replication motile males fertilize female gametes to form zygotes. *Pf* s48/45 is expressed on the surface of both male and female gametes where it is bound to the plasma membrane through a GPI-anchor (5) and forms a stable complex with *Pf* s230 (6). Both *Pf* s48/45 and *Pf* s230 are important for male fertility (7).

Humans develop naturally acquired immunity against *P. falciparum* gametocytes (8–10) and antibodies against *Pf* s230 and *Pf* s48/45 have been associated with TB activity in some but not all immune epidemiological studies (11, 12). Recently, we demonstrated that *Pf* s48/45- and *Pf* s230-specific antibodies exhibit strong TB activity in the standard membrane feeding assay (SMFA) (13), the gold standard for assessing transmission blockade *ex vivo* (2, 14–16). Whether such antibodies act synergistically, as observed for combinations of mAbs targeting different epitopes on *Pf* s48/45 (2), is not yet known.

*Pf* s48/45 and *Pf* s230 are members of the six-cysteine (6-Cys) s48/45 protein family and contain three and fourteen 6-Cys domains respectively (17). Each 6-Cys domain contains up to six cysteine residues that are involved in intra-domain disulfide bond formation which results in conformational antibody epitopes. The C-terminal 6-Cys domain of *Pfs*48/45 contains the conformational epitope I, which is targeted by the most potent TB monoclonal antibody described to date, mAb45.1 (18). We have recently used the *Lactococcus lactis* expression system for the production of the C-terminal 6-Cys domain of *Pf* s48/45 (6C) as a fusion protein (R0.6C) with the N-terminal GLURP-R0 region (19, 20). The resulting fusion protein can be produced in high yields of properly folded monomeric protein which elicited high levels of TB antibodies in small rodents (19, 20). In the case of *Pf* s230, the C fragment spanning the N-terminal pro-domain and first three 6-Cys domains has been shown to elicit the most potent TB antibodies (21). The presence of three 6-Cys domains suggests that disulfide bonds may be critical for proper folding of each of these domains. Accordingly, a series of *Pf* s230-specific transmission-blocking monoclonal antibodies did not recognize reduced *Pf* s230 (22). In an attempt to identify the minimal *Pf* s230-domain involved in the generation of TB antibodies, *Pf* s230 constructs containing the Pro, Pro+I, Pro+I,II, and Pro+I,II,III domains were produced individually in the wheat germ cell-free system (23). Interestingly, the N-terminal Pro domain, which lacks cysteines, was sufficient to elicit complement-dependent TB activity in the SMFA, suggesting that TB antibodies may also be directed against non-conformational epitopes (23). With respect to *Pf* s230, the C-fragment was the first construct to elicit TB antibodies; however, oocyst reduction was incomplete suggesting that folding was compromised by an incorrect cysteine connectivity (21). Another construct, *Pf* s230D1, corresponding to amino acid residues 444 to 736 was produced in *Pichia pastoris* as a properly folded protein and elicited TB antibodies in rodents (24).

While clinical trials with *Pf* s230D1 are ongoing (ClinicalTrials.gov Identifier: NCT02334462) and R0.6C is in early clinical development phase, we sought to identify more potent *Pf* s48/45- and *Pf* s230-based immunogens. We hypothesized that a combination of antibodies against both proteins would be more potent than against each antigen individually. Therefore we constructed chimeric proteins composed of fragments of both *Pf* s230 and *Pf* s48/45, expressed these in *L. lactis* and evaluated antibody responses in rodents. A multicomponent hybrid protein containing both *Pf* s48/45 and *Pf* s230 holds the potential to lower the required threshold of functional antibodies and to reduce the risk of escape mutations.

## Methods

### Preparation of Constructs

Three different truncated forms of *Pf* s230 from N-terminus, i.e. Pro (pro domain AA 443 to 590), Pro+I (pro domain and domain I, AA 443 to 736) and Pro+I,II,III (pro domain through domain III, AA 443 to 1132) were amplified by PCR from *P*. *falciparum* 3D7 DNA (GenBank accession number L08135) and cloned into the *Bgl*II restriction site of pSS5 plasmid containing N-terminus Spycatcher (25). *Pf* s48/45_291−428_ (6C) was amplified from an expression vector encoding R0.6C (19, 20) using the forward primer 5′-CCATGGATCCGAAAAAAAAGTCATACACGGATGTAACTTC-3′ and the reverse primer 5′-CCATAGATCTTGCTGAATCTATAGTAACTGTCATATAAGC-3′. The amplified PCR product was digested with *Bam*HI and *Bgl*II (underlined) and cloned in frame into plasmids containing the Pro or Pro+I inserts to generate Pro-6C and Pro+I-6C fusion constructs, respectively. All the constructs were verified by DNA sequencing and transformed into *L. lactis* MG1363 by electroporation for expression of recombinant proteins with 6xHis tags.

### Fermentation and Protein Purification

Fermentation of *L. lactis* MG1363, containing *Pf* s230 or *Pf* s230-*Pf* s48/45 fusion constructs were carried out as described previously (19, 26). Briefly, cell-free culture-filtrates were concentrated five-fold and buffer exchanged into Tris buffer (50 mM Tris, 50mM NaCl pH 8.0 supplemented with 10mM Imidazole) using a Quix Stand Benchtop system (Hollow fiber cartridge with cutoff at 10,000 or 30,000 Da, surface area 650cm^2^, GE Healthcare, Sweden) followed by filtration through a Durapore filter (PVDF, 0.22 μm, Millipore) and applied to a 5 ml HisTrap HP column (GE Healthcare, Sweden). Bound protein was eluted with 500mM Imidazole in Tris buffer pH 8.0 (50 mM Tris, 50mM NaCl) at a flow rate of 4 ml/min. Fractions containing the desired protein were further applied to a 5 ml HiTrap Q HP column (GE Healthcare, Sweden) for purification of monomeric proteins. Bound protein was eluted through step gradient elution in Tris buffer pH 8.0 (50 mM Tris, 1mM EDTA, 1 M NaCl) and fractions containing monomers were concentrated by a VIVA spin column with a 10 or 30 kDa cutoff (Vivascience, Germany), and kept in 50 mM Tris, 250 mM NaCl and 1 mM EDTA, pH 8.0 at −80°C until use. Immune purification for Pro-6C and Pro+I-6C was done as previously described (26). Fractions containing the desired protein were pooled and then concentrated and buffer exchanged against 50 mM Tris, 100 mM NaCl, and 1 mM EDTA, pH 8.0 and kept at −80°C until use. Fractions were analyzed by SDS-PAGE and immune blotting with mAb45.1 against *Pf* s48/45 conformational epitope I. Protein concentrations were measured using a BCA kit (Thermo Fisher Scientific, USA).

### Protein Characterization

Analytical size exclusion high-performance liquid chromatography (SE-HPLC) of purified fusion proteins was performed as described previously (19, 20). Briefly, 5 μl of protein was loaded on an Agilent advance Bio SEC 300 Å, 2.7 μm, 4.6 × 300 mm SEC column (Agilent Technologies, GB) and eluted with a 0.1 ml/min flow of elution buffer (phosphate buffer) at room temperature. Protein standards (Sigma Aldrich) were also run using the same conditions mentioned above for sizing of the purified recombinant proteins. The amount of free cysteine residues was measured using Ellman's Reagent (Thermo Fisher Scientific, USA) following the manufacturer's instructions. A standard curve was constructed using known concentrations of free cysteine (Sigma-Aldrich, USA). Folding was determined in the mAb45.1 sandwich ELISA as described (19, 26).

### Production of VLPs

SpyTag was genetically fused to the N-terminus of AP205, as previously described (27). In brief, the SpyTag peptide sequence (AHIVMVDAYKPTK) was fused to the gene sequence encoding the major AP205 coat protein (Gene ID: 956335) using a flexible linker (GSGTAGGGSGS) between the two sequences. The SpyTag-AP205 VLPs were expressed in *Escherichia coli* One Shot® BL21 Star™ (DE3) cells (Thermo Fisher Scientific, USA) and purified by ultracentrifugation using an Optiprep™ (Sigma-Aldrich, USA) gradient. For conjugation to VLPs, purified soluble Pro-6C or Pro+I-6C proteins were incubated at a molar ratio of 1:1 (VLP/antigen) in a 1xPBS buffer for 2 h at room temperature. Unbound protein was removed by dialysis against PBS using 1,000 MWCO dialysis tubing (Spectrum Labs, USA). Densitometric analysis of SDS-PAGE gels was used to estimate protein concentrations.

### Dynamic Light Scattering

Uncoupled VLP, soluble proteins and proteins conjugated to VLP were adjusted to 0.5–1 mg/ml in PBS and spun at 15,000 g for 10 min. Seventy Microliter sample was loaded into a disposable Eppendorf Uvette cuvette (Sigma-Aldrich, USA) and measured at 25°C on a DynoPro NanoStar (WYATT Technology, USA) equipped with a 658 nm laser. Each sample was measured 20 times and intensity-average size and percentage polydispersity (PD) was estimated using Dynamic software (Version 7.5.0).

### Electron Microscopy

Pro-6C or Pro+I-6C coupled to VLP (with concentrations between 0.4 and 0.5 mg/ml based on antigen content) were incubated on carbon-coated and glow-discharged grids and negatively stained with 2% phosphotungstic acid (pH 7.4). The particles were analyzed on a CM 100 BioTWIN electron microscope with an accelerating voltage of 80 kV. Images were acquired using an Olympus Veleta camera and particle size was estimated using iTEM software.

### Animals and Immunogenicity Studies

In the first experiment, groups (*n* = 5) of CD-1 mice 5–7 weeks of age (Janvier Labs, Denmark) were immunized 3 times at 3-week interval by the intramuscular injection of equimolar amounts of immune-purified Pro-6C and the individual *Pf* s230 and *P*fs48/45 recombinant protein constructs formulated with Alhydrogel® (Brenntag, Denmark) to a final concentration of 2 mg/ml Aluminum. Please note that six mice received R0.6C. Each dose contained 128 pmoles of soluble protein (equivalent to 2 μg 6C). Serum was collected on days 14, 35, and 56. In the second experiment, groups (*n* = 8) of CD-1 mice were immunized with 64 pmoles (equivalent to 1 μg 6C) Pro-6C or Pro+I-6C (soluble or conjugated to VLP) as described above for the first experiment. One mouse receiving Pro+I-6C was terminated due to behavioral abnormalities not related to vaccination. All animals were treated in accordance with the regulations and guidelines of the European and National authorities.

### Enzyme-Linked Immunosorbent Assay (ELISA) for Antibody Response Measurement

Gametocyte extract ELISA was performed with cultured sexual stage of *Pf* NF54 parasites as previously described (26). Serum immunoglobulin (IgG) subclass levels were measured using ELISA as previously described (28). For antigen-specific ELISA, 96-well plates (Nunc MaxiSorp) were coated with 0.5 μg/well of *Pf* s48/45-6C (25), Pro+I, or Pro+I,II,II as appropriate. Antigen-specific antibodies were detected using HRP-conjugated polyclonal goat anti-mouse IgG (Novex A16072, diluted 1:3000). Antibody midpoint titer (EC50) was calculated using sigmoidal curve fitting. One-sided analysis of variance on the log-transformed values was used to confirm that ELISA data contain essential differences. Mann-Whitney test was then used to investigate whether the chimera elicited higher levels of specific antibodies compared to individual components. *p*-Values are two-sided and quoted without adjustment for multiple testing since the significance of the chimera was the primary problem under investigation. *p*-values ≤ 0.05 were considered significant. Statistical analysis was conducted using GraphPad Prism 7 (GraphPad Software, USA).

### Standard Membrane Feeding Assay (SMFA)

The biological activity of specific antisera was assessed in the SMFA as previously described (26, 29). Depending on availability, wild type *P. falciparum* NF54 gametocytes or transgenic *P. falciparum* NF54 (NF54-HGL) gametocytes expressing luciferase (29) were fed to *Anopheles stephensi* mosquitoes that were reared and maintained at Radboudumc, The Netherlands. Non-heat inactivated mice sera, and active or heat-inactivated human complement was added to the cultured material prior to feeding to mosquitoes. After 6–8 days, oocysts in 20 fed mosquitoes were counted by microscopy, or quantified in four pools of five mosquitoes (NF54-HGL) by measuring luciferase levels (29). Samples were tested in two independent SMFA experiments. Luminescence-based TRA estimates from two independent feeds with each of four pools of five mosquitoes were made using generalized linear mixed models (GLMMs) without zero-inflated negative binomial error structure (30, 31). Microscopy-based estimates from two feeds with 20 mosquitoes each were made using GLMMs with zero-inflated negative binomial error structure (30, 31). Statistical differences between test samples were determined using General Linear Mixed Regression analysis. Statistical analyses were performed using R studio (v. 3.2.4, The R Foundation, Boston, USA). Pre-immune pooled mice serum samples were also tested in a single independent SMFA experiment (Figure S2).

### Depletion of Antigen-Specific IgGs

*Pf* s230- and *Pf* s48/45-specific antibodies were depleted from serum using *Pf* s230-CMB (32) and R0.10C-containing columns respectively, as previously described (13). The *Pf* s230-CMB fragment contains AA 444-730 and thus covers the Pro+I fragment expressed in *L. lactis*. To test if all antigen-specific IgGs were depleted, the flow through serum (depleted serum) was tested in ELISA using plates coated with *Pf* s230-CMB or SpyC-6C as appropriate (25, 26).

## Results

### Expression of a Multivalent *Pf*s230-*Pf*s48/45 Chimera in *L. Lactis*

**Figure 1:**
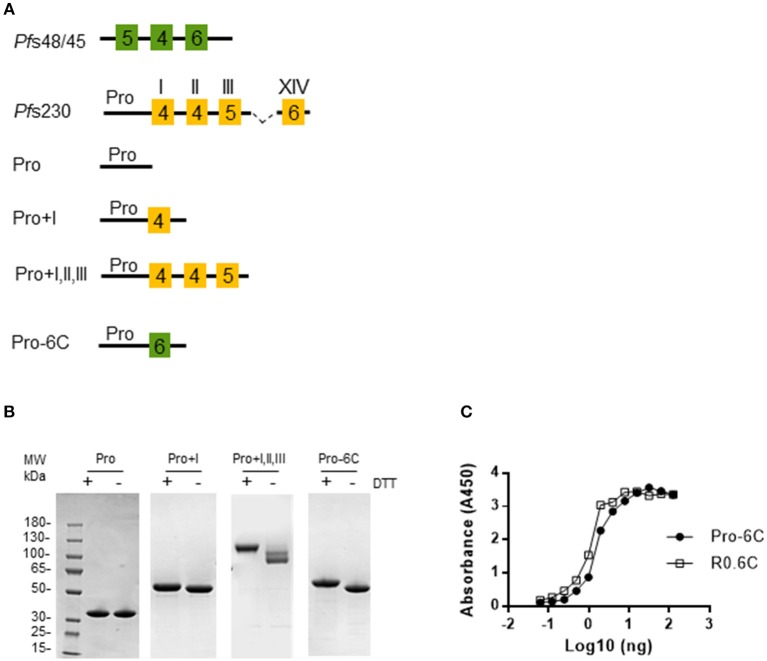
Production of recombinant *Pf*s230 and *Pf*s48/45. **(A)** Schematic representation of *Pf*s230 constructs and *Pf*s230-*Pf*s48/45 chimera. Each construct contains the SpyCatcher sequence at the N-terminus and a His-tag at the C-terminus. 6-Cys domains are shown as boxes and numbers indicate the number of cysteines. The Pro-domain does not contain cysteines. **(B)** Coomassie blue stained 4–12.5% polyacrylamide gel of conventionally purified *Pf*s230 constructs and immune-purified Pro-6C chimera. Protein was loaded in each lane with (+) or without (–) DTT (10 mM). The sizes (kDa) of the molecular mass markers are indicated. **(C)** Sandwich ELISA of purified Pro-6C chimera. The antigens were captured with mAb45.1 and detected with anti-His-HRP. Immune purified R0.6C were used as a reference. X-axis is shown on a logarithmic scale.

To test whether a multivalent vaccine targeting *Pf* s48/45 and *Pf* s230 is immunogenic, we generated a chimeric construct containing the Pro domain of *Pf* s230 fused to the 6C fragment of *Pf* s48/45 (Figure 1A). We anticipated that this Pro-6C fusion protein would express well in *L. lactis* since the *Pf* s230-Pro domain is glutamate-rich, does not contain cysteines, and is similar to the R0 domain which previously enhanced expression of properly-folded 6C in *L. lactis* (25, 26). In addition to Pro-6C, we made constructs that either contained *Pf* s48/45 or *Pf* s230 fragments (Figure 1A). *L. lactis* MG1363 harboring these constructs were grown in a 1L bioreactor and the respective recombinant proteins were purified from the clarified supernatant through the C-terminal His-tag by immobilized metal affinity chromatography and ion exchange chromatography (Figure 1B). As expected, protein yields decreased with increasing number of *Pf* s230 domains (Table S1). Pro-6C was further immune-purified on a mAb 45.1-column to enrich for properly folded protein species (Figure 1B). The yield of immune-purified Pro-6C was 15 mg/L, similar to that of R0.6C. Conformational mAb 45.1 against the *Pf* s48/45 epitope I reacted with Pro-6C and this binding was equivalent to that of immune-purified R0.6C, suggesting that they exhibit similar cysteine-connectivity (Figure 1C).

### Immunogenicity of Soluble *Pf*s48/45 and *Pf*s230 Protein Constructs

**Figure 2:**
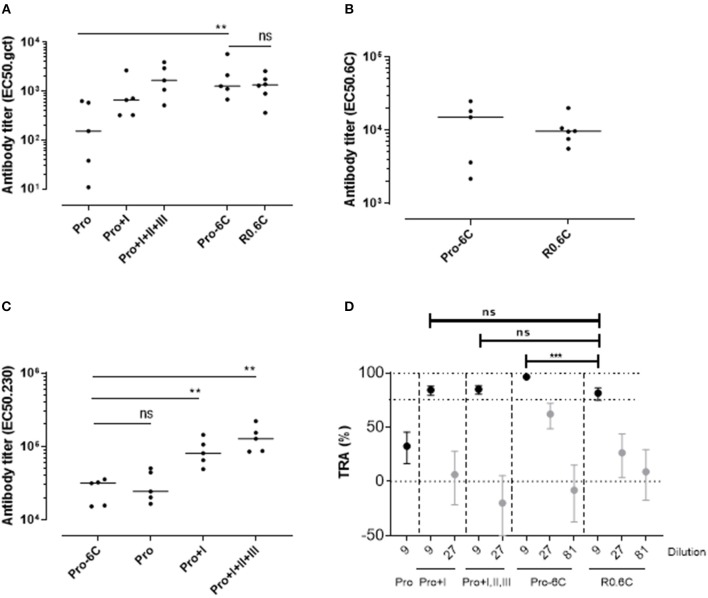
Immunogenicity of Pro-6C and individual fragments. Groups of mice (*n* = 5) were immunized with immune purified Pro-6C in a comparative study with individual Pro, Pro+I, Pro+I,II,III and R0.6C constructs. Day 56 serum was tested for antibody reactivity on ELISA plates coated with **(A)** gametocyte extract, **(B)** *Pf*s48/45-6C or **(C)** *Pf*s230 Pro+I,II,III. Antibody titers (dots represent individual mice) are expressed as EC50 values. Horizontal lines represent median values. The asterisks represent statistical significance determined by Mann-Whitney test (**p < 0.01, ns not significant). **(D)** Functional activity of serial dilutions of pooled sera in the SMFA. Transmission reducing activity (TRA) is the reduction of oocyst numbers compared to a serum control. All samples were tested in the presence of active complement. Data points are best estimates of two independent SMFA experiments and error bars represent 95% confidence intervals. Comparison between test samples was done using General Linear Mixed Regression analysis (***p < 0.005, ns, not significant).

Groups of mice were immunized 3 times at 3-week interval with equimolar amounts of Pro-6C and individual *Pf* s230 and *Pf* s48/45 recombinant protein constructs formulated on Alhydrogel®. We used a suboptimal antigen dose to detect differences in immunogenic properties between protein constructs. Chimeric Pro-6C elicited significantly higher levels of gametocyte-specific antibodies than those obtained with the individual Pro domain (Mann-Whitney test, *p* = 0.0079) and levels comparable to those obtained with Pro+I, and R0.6C (Figure 2A). We found that levels of specific antibodies against the Pro and 6C domains were similar in mice immunized with Pro-6C compared to mice immunized with the individual Pro and 6C (R0.6C) antigens, suggesting that these domains do not exhibit antigenic competition (Figures 2B,C). As expected, levels of *Pf* s230-specific antibodies increased with *Pf* s230 fragment length (Figure 2C). The functional activity of vaccine-induced antibodies was determined by testing pooled antisera from each group in serial dilutions in the SMFA. All proteins except the Pro domain, elicited a TB response of >80% at a 1/9 dilution. Interestingly, Pro-6C induced higher levels of functional antibodies than the other recombinant proteins including R0.6C (*p* < 0.001) (Figure 2D), supporting further investigation of multi-component *Pf* s230-*Pf* s48/45 vaccines.

### Generation of Soluble and VLP-Based Chimeric Constructs

**Figure 3:**
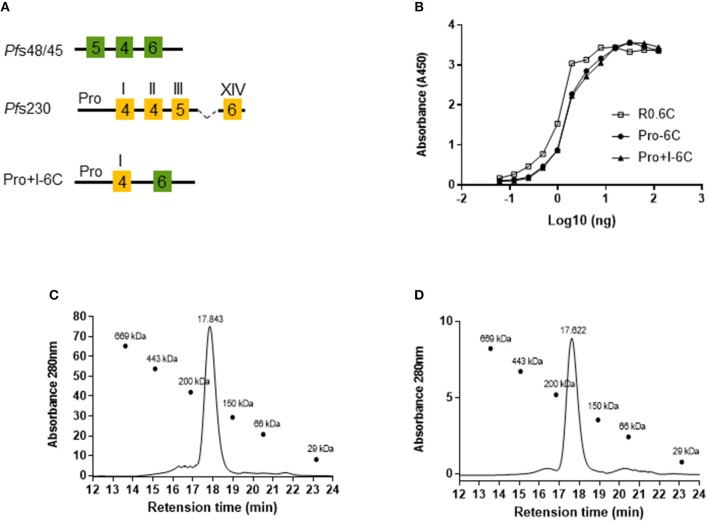
Design and characterization of a multi-domain *Pf*s230-*Pf*s48/45 chimera. **(A)** Schematic representation of the Pro+I-6C chimera. This protein contains the SpyCatcher sequence at the N-terminus and a His-tag at the C-terminus. 6-Cys domains are shown as boxes and numbers indicate the number of cysteines. The Pro-domain does not contain cysteines. **(B)** Sandwich ELISA of purified chimera. Immune purified R0.6C was used as a reference. Size exclusion chromatography analysis of **(C)** Pro-6C and **(D)** Pro+I-6C. SE-HPLC was performed under native conditions in a phosphate buffer of pH 7.2 to determine the amount of monomer in the sample. The sizes (kDa) of the molecular mass markers are indicated.

**Figure 4:**
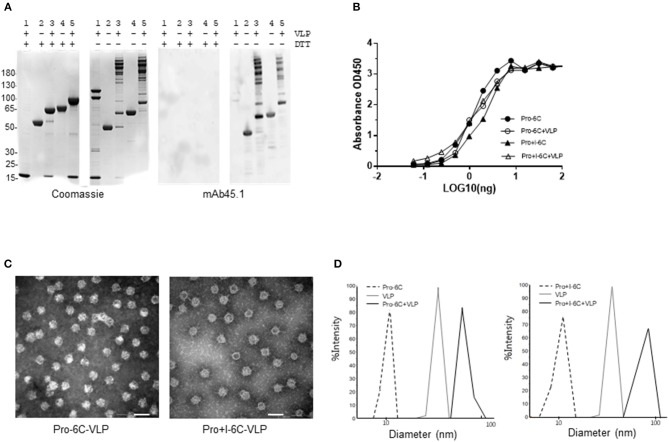
Characterization of virus-like particle-based vaccines. SpyCatcher tagged Pro-6C and Pro+I-6C were mixed with SpyTag-AP205 resulting in a unidirectional display of the chimeras on the VLP surface. **(A)** Reduced and non-reduced SDS-PAGE gel and western blot. The gels (left) are stained with coomassie blue, while the western blots (right) are developed with mAb45.1 as primary antibody. The following was loaded corresponding to the numbers; Lane 1: AP205 (VLP); lane 2: Pro-6C; lane 3: Pro-6C-VLP; lane 4: Pro+I-6C; Lane 5: Pro+I-6C-VLP. **(B)** Sandwich ELISA, using mAb 45.1 as the solid phase capture antibody. **(C)** Transmission electron microscopy images (negative stain) of the VLP-based vaccines after assembly. Both Pro-6C-VLPs and Pro+I-6C-VLPs appear non-aggregated, uniformly dispersed and have an estimated size of 30 nm. Scale bar 100 nm. **(D)** Dynamic light scattering (DLS) profile of the vaccine components Pro-6C [10.5 nm, polydispersity (PD) 10.7%], Pro+I-6C (10.8 nm, PD 20.7%), VLP (25.6 nm, PD 16.8%) and the purified vaccine products; Pro-6C-VLP (71.8 nm, PD 11.5%), and Pro+I-6C-VLP (73.7 nm, PD 15.8%).

Next, we tested whether the potency of Pro-6C could be further increased by including the first 6-Cys domain of *Pf* s230 (Pro+I-6C) (Figure 3A). The Pro+I-6C chimera was purified following the same workflow developed for Pro-6C. The yield of immune-purified Pro+I-6C was 5 mg/L, which was 3-fold lower than that of Pro-6C most likely due to the additional cysteine residues (Table S1). The folding of both chimera was similar as determined in the mAb45.1 sandwich ELISA (Figure 3B). Disulfide-bonding was confirmed by demonstrating very low levels of free thiol groups (<1%) under native conditions (data not shown). Immune purified Pro-6C and Pro+I-6C eluted as single peaks by analytical size exclusion chromatography demonstrating that they form homogeneous solutions of monomeric protein species (Figures 3C,D). Before starting *in vivo* immunogenicity studies with both soluble constructs, we also coupled Pro-6C and Pro+I-6C to virus-like particles (VLPs) to see if this would further increase the immunogenicity of the chimeras. Both Pro-6C and Pro+I-6C contained a SpyCatcher domain allowing covalent coupling to SpyTag-decorated AP205 VLPs (25, 33). Spy-Catcher Pro-6C and Pro+I-6C coupled to SpyTag VLPs efficiently (Figure 4A) and properly folded *Pf* s48/45 epitope I was retained during conjugation, as shown by western blot and mAb45.1 sandwich ELISA (Figures 4A,B). Both VLPs formed homogenous populations of non-aggregated antigen-VLP complexes as demonstrated by transmission electron microscopy (Figure 4C). Furthermore, the VLP-particles displaying Pro-6C and Pro+I-6C demonstrated a low percentage of polydispersity (<16%) measured by dynamic light scattering (DLS) experiments and an average size of 71.8 and 73.7 nm, respectively (Figure 4D).

### Immunogenicity of Soluble and VLP-Based Chimeric Constructs

**Figure 5:**
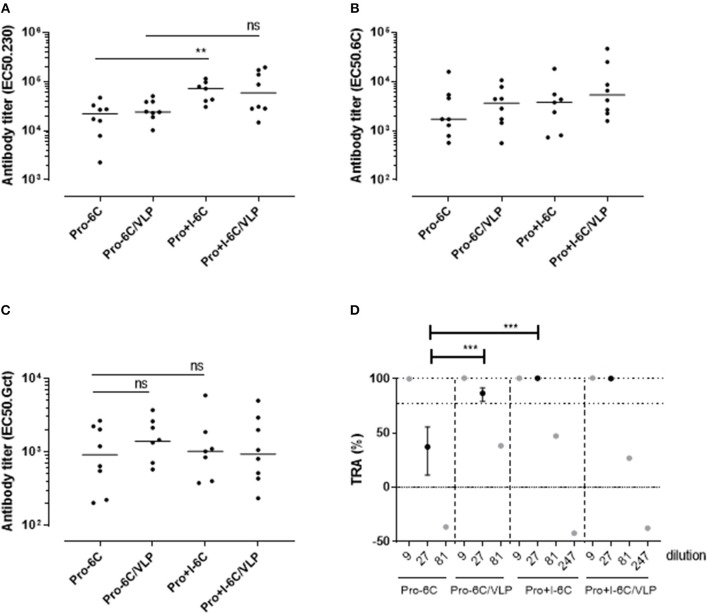
VLP-delivery of Pro-6C and Pro+I-6C. Groups of mice (*n* = 8) were immunized with soluble Pro-6C and Pro+I-6C or bound to AP205. Day 56 serum was tested for antibody reactivity on ELISA plates coated with **(A)** *Pf*s230 Pro+I,II,III **(B)**, *Pf*s48/45-6C or **(C)** gametocyte extract. Antibody titers (dots represents individual mice) are expressed as EC_50_ values. Horizontal lines represent median values. The asterisks represent statistical significance determined by Mann-Whitney test (**p < 0.01, ns not significant). **(D)** Functional activities of serial diluted sera were assessed in the SMFA. Transmission reducing activity (TRA) is the reduction of oocyst load compared to a serum control. All samples are tested in the presence of active complement. Data points are best estimates of two independent SMFA experiments and error bars represent 95% confidence intervals. Note that 1/81 and 1/247 samples were tested in SMFA only once and therefore no confidence intervals are given. Comparison between samples was done using General Linear Mixed Regression analysis (***p < 0.005, ns, not significant).

The immunogenicity of soluble Pro-6C and Pro+I-6C, and of Pro-6C and Pro+I-6C coupled to VLPs was assessed *in vivo*. Groups of CD-1 mice (*n* = 8) were immunized 3 times at 3-week intervals with equimolar amounts of antigen adjuvanted on Alhydrogel®. Soluble Pro+I-6C elicited significantly (Mann-Whitney test, *p* = 0.0079) higher levels of *Pf* s230-specific responses than soluble Pro-6C (Figure 5A). However, this increase was not associated with higher levels of gametocyte-specific antibodies (Figure 5C), possibly due to the difference in Pfs230-specific antibody levels being masked by higher 6C-specific signals in the gametocyte ELISA, as observed for single antigen constructs (Figure 2A). The functional activity of pooled anti-sera from each group was then tested at serial dilutions in the SMFA. Antibodies against soluble Pro+I-6C promoted higher SMFA activity than antibodies against Pro-6C at a 1/27 dilution (*p* < 0.001) (Figure 5D), in line with the SMFA results obtained with the single domain constructs (Figure 2D). VLP display did not provide an increase in gametocyte-, *Pf* s48/45-, or *Pf* s230-specific antibodies for both Pro-6C and Pro+I-6C (Figures 5A–C). VLP-display of Pro-6C enhanced (Mann-Whitney test, *p* < 0.001) the production of functional antibodies as demonstrated in the SMFA while there was no such effect for Pro+I-6C (Figure 5D).

### Functional Activity of Domain Specific Antibodies in the SMFA

**Figure 6:**
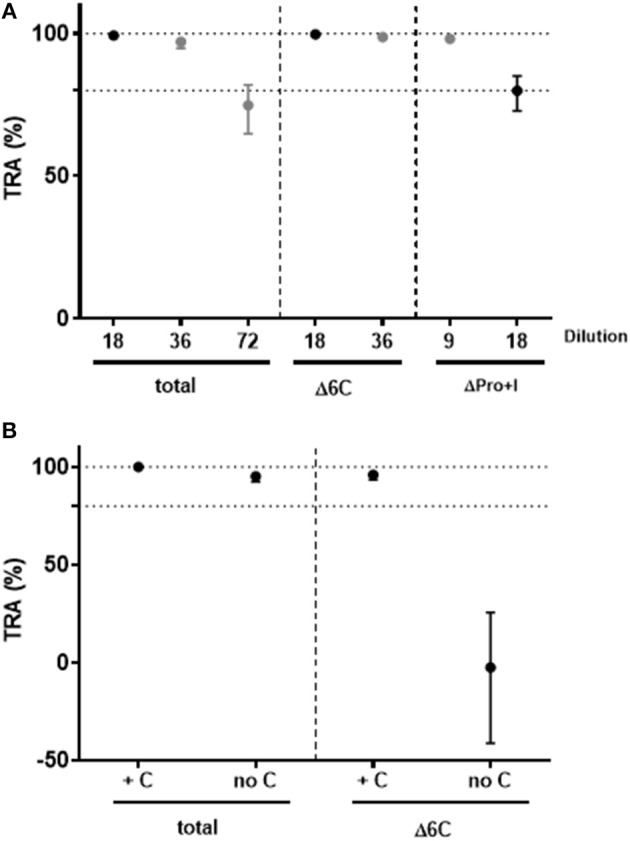
Functional activity of domain-specific antibodies generated by Pro+I-6C. Domain-specific antibodies were depleted from pooled sera of mice immunized with Pro+I-6C, using columns containing *Pf*s230-Pro+I and *Pf*s48/45-10C (13), to generate sera that recognize either 6C or Pro+I respectively. **(A)** Serial dilutions of sera containing Pro+I antibodies only (Δ6C) and sera containing 6C antibodies only (ΔPro+I) were tested in the SMFA, in the presence of active complement. **(B)** Total sera and sera containing Pro+I antibodies only were re-tested with (+C) and without (no C) active complement at a dilution of 1/9. Transmission reducing activity (TRA) is the reduction of oocyst numbers compared to a serum control. Data points are best estimates of two independent SMFA experiments and error bars represent 95% confidence intervals.

To investigate the functional activity of domain-specific antibodies, pooled sera from mice immunized with soluble Pro+I-6C (no VLP) were depleted for *Pf* s230- and *Pf* s48/45-specific antibodies using affinity columns with immobilized *Pf* s230 and *Pf* s48/45 respectively, as previously described for human antibodies (13). Antibody depletion was confirmed by ELISA (data not shown). The functional activity of antibody-depleted sera was then tested in the SMFA in 2-fold serial dilutions in the presence of complement. Sera depleted of *Pf* s230-specific or *Pf* s48/45-specific antibodies retained transmission blocking activity at 9- and 18-fold dilutions, respectively, demonstrating that functional antibodies against both proteins are induced by the Pro+I-6C construct (Figure 6A). Since the TB activity of *Pf* s230-specific antibodies depends on complement (22), depleted anti-sera were re-tested in SMFA with and without active complement. As expected, *Pf* s230-specific antibodies (Δ6C) lost their functional activity in the absence of active complement (Figure 6B). Since complement fixation is dependent on specific antibody subclasses (34), we determined the IgG subclass profile elicited by the Pro+I-6C formulation. The chimera elicited predominantly IgG1 antibodies, and to a lower extent IgG2a and IgG2b antibodies (Figure S1). Altogether, these data show that functional antibodies are generated against both domains of the chimeric construct and that *Pf* s230-specific antibodies are complement dependent.

## Discussion

*Pf* s230 and *Pf* s48/45 are expressed during the *P. falciparum* sexual stages in humans and elicit antibodies which effectively prevent parasite multiplication in the infected mosquito (13). Promising *Pf* s230- and *Pf* s48/45-based MTBV candidates are currently entering clinical development individually. Here we set out to produce constructs with higher potency and to this end produced and tested chimeric proteins based on both vaccine candidates. To the best of our knowledge, this is the first study that explores a multivalent MTBV based on *Pf* s230 and *Pf* s48/45. Our chimeric proteins induce antibodies that have >80% transmission reducing activity in the SMFA, which is high enough to meet the no/go decision criterion for selection and further vaccine development (35). Overall, our data show that these chimeric proteins elicited antibodies with higher TB activity in the SMFA than the single proteins alone (Figures 2D, 5D) and are therefore attractive next generation vaccine candidates.

One concern when generating multivalent vaccines is that one of the components is immunodominant and that responses against the other component are therefore compromised (36, 37). To investigate this we tested specific antibody responses against the individual domains by ELISA and demonstrated that these are not affected when the domains are presented as part of chimeric proteins. Moreover, the depletion experiments showed that antibodies against the Pro+I- and 6C-domains are functional in the SMFA. Interestingly, the Pro+I-6C chimera elicited higher levels of functional *Pf* s230-specific antibodies than the Pro+I domain alone; sera from Pro+I immunized mice showed no TRA in the SMFA at 1/27 dilution, whereas sera depleted of 6C-specific antibodies from mice immunized with Pro+I-6C still retained 99% TRA at 1/36 dilution. This apparent difference was not reflected in levels of specific antibodies detected in the *Pf* s230-ELISA indicating that functional activity in the SMFA is not only dependent on quantity but also the quality of antibodies. Importantly, there was no difference in levels of functional antibodies against the *Pf* s48/45-6C-domain when comparing Pro+I-6C and R0.6C immunized mice suggesting that the increase in functional activity of *Pf* s230-specific antibodies is, at least in part, related to a better presentation of antibody epitopes in the *Pf* s230 domain I of the Pro+I-6C chimera.

Adjuvants with the ability to enhance antigen immunogenicity are critical components of an efficacious subunit vaccine. In the case of chimeras that include *Pf* s230, it is particularly important that vaccination elicit antibodies with complement-fixing activity. Here we show that Pro+I-6C formulated on Alhydrogel® elicited high levels of IgG1 antibodies, but also Pro+I specific IgG2a and IgG2b antibodies in mice. Murine IgG2a and IgG2b are subclasses of IgG that bind with high affinity to human complement (34). The activation of complement may subsequently trigger lysis of gametes in the infected mosquito (22). It remains to be determined whether the Pro+I-6C chimera may also elicit complement-fixing antibodies in humans.

Both *Pf* s230- and *Pf* s48/45-based vaccine constructs are rich in cysteine and proper disulfide bond formation is critical for functional antibody responses (22, 38). Therefore, successful construction and production of chimeric proteins depend on the maintenance of conformational integrity of immunologically relevant regions of the individual domains. The mAb45.1 is a conformational mAb (2) that reacts with properly folded *Pf* s48/45 but not disulphide reduced *Pfs*48/45 (38). Its reactivity with the fusion proteins indicate proper cysteine connectivity of the *Pf* s48/45 domain. Accordingly, our results from ELISA and immunoblotting analysis showed that the purified chimeras expressed in *L. lactis* were strongly recognized by mAb45.1 and had thus retained conformational epitope I in the *Pf* s48/45-6C domain. It was particularly encouraging that recombinant Pro+I-6C reacted with mAb45.1 since *Pf* s230 domain I contains four cysteine residues which may potentially interfere with disulfide bonding of the *Pf* s48/45-6C domain. While disulphide bond studies have not been completed on the two chimera, the positive reactivity with mAb45.1, indicate that the *Pf* s230 sequence does not disrupt the proper disulphide formation of the *Pf* s48/45 6C domain contained within. Correct folding was further supported by antibody depletion experiments showing that both domains of the chimeras elicited functional antibodies. The data thus demonstrate that *L. lactis* is not only suitable for expression of *Pf* s48/45 fragments (19), but also for *Pf* s230 fragments and fusions thereof. The immune purification of the two chimeras used here is not compliant with cGMP manufacturing since a monoclonal antibody of rat origin was used. However, a purification process based on conventional chromatographic procedures is currently being developed. Although yields and purity remain to be determined, it is likely that high product yields can be obtained through upscaling the fermentation process which is straightforward since there is no requirement for oxygen and vigorous stirring during fermentation (39). Additionally, yields of properly folded protein species may also be increased through protein refolding processes as those developed for R0.6C (20).

In conclusion, we have produced two chimeras composed of leading vaccine candidates against the transmission stages of *P. falciparum*. Both chimera elicited high levels of functional antibodies in rodents and outperformed the corresponding individual protein fragments. Previously, rodents have been immunized with *Pf* s25 administered together with either *Pf* s28 or *Pf* s230C (40). In contrast to our findings for Pfs48/45-Pfs230 chimeras, the *Pf* s25-based dual-antigen vaccines did not elicit higher levels of functional antibodies than the corresponding single antigen vaccines (40). Together these data demonstrate that additive effects can only be achieved for certain antigen combinations. Our results do not only support the use of chimeric proteins for MTBV development but also for malaria vaccine development in general (26, 28, 41, 42). Another advantage of multi-component vaccines is that the production of antibodies against multiple antigens might help reduce the spread of potential escape mutants in the population. Such escape mutants may compromise overall vaccine efficacy as exemplified with the RTS,S malaria vaccine (43). This multivalent strategy is thus conceptually attractive and will constitute a novel means toward control and eventually eradication of malaria once clinical efficacy has been demonstrated.

## Ethics Statement

The animal studies were approved by the Danish Animal Experiments Inspectorate. Approval number: 2013-15-2934-00902/BES.

## Author Contributions

SS, ST, BC, KT, RS, WG, G-JvG, MvdV-B, and MJ: performed the experiments. ST, MN, AS, and AFS: designed the VLP constructs. SS, MT, and MJ: designed the experiments. MT, MJ, and RWS: wrote the manuscript. All authors reviewed the manuscript.

### Conflict of Interest Statement

A patent application covering the VLP spy-technology has been filed by the University of Copenhagen. The authors declare that the research was conducted in the absence of any commercial or financial relationships that could be construed as a potential conflict of interest.

## Supplementary Material

Figure S1 Subclass-specific antibody responses in mice immunized with Pro+I-6C. Day 56 sera were tested for antibody reactivity on ELISA plates coated **(A)** Pro+I or **(B)** 6C. Results for individual mice are shown, and a horizontal bar represents each median value.

Figure S2 Pre-immune mouse serum contains low intrinsic TRA. Pooled mouse serum from each group was tested at 1/9 dilution in a single SMFA experiment. TRA is the reduction of oocyst numbers compared to a serum control. Error bars represent 95% confidence intervals. TRA values below 50% have large confidence intervals and this can explain the observed variation between different groups. The mean TRA of all groups is 21% (CIs: 5.7–34.1%) demonstrating that mouse serum has low intrinsic TRA.

Table S1 Production and quantification of *Pf*s230 and *Pf*s230/*Pf*s48/45 chimeric protein in *L. lactis*.
