# Supplementary figures and images for: Pfs230 and Pfs48/45 Fusion Proteins Elicit Strong Transmission-Blocking Antibody Responses Against Plasmodium falciparum
Source: Front Immunol. 2019 Jun 5;10:1256. doi: 10.3389/fimmu.2019.01256 (PMC6560166; doi:10.3389/fimmu.2019.01256)

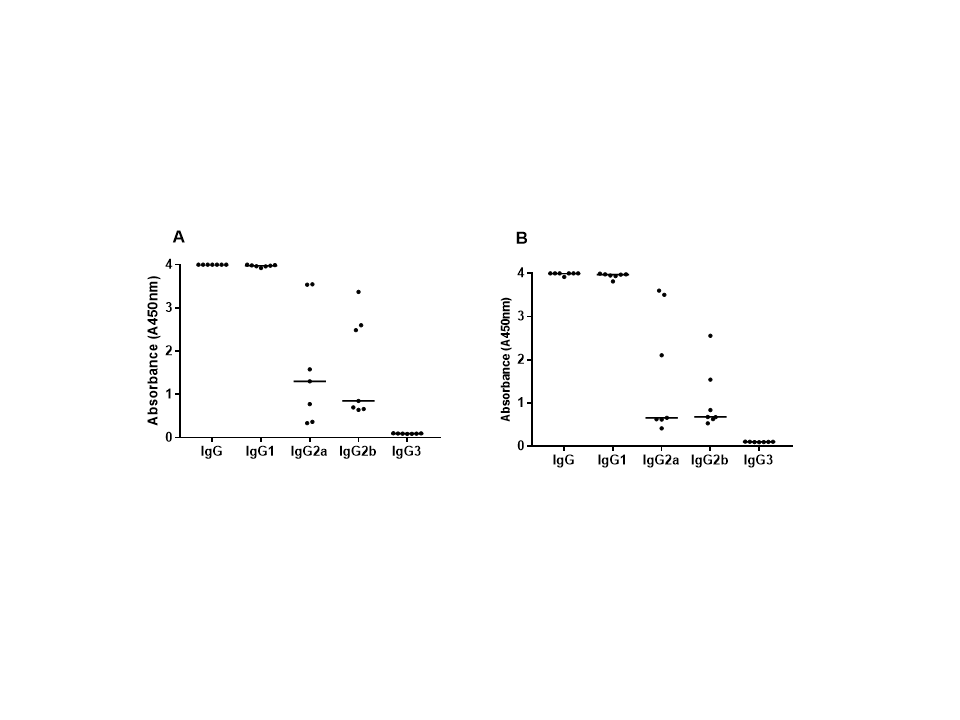

Supplement: Figure S1 — Subclass-specific antibody responses in mice immunized with Pro+I-6C. Day 56 sera were tested for antibody reactivity on ELISA plates coated (A) Pro+I or (B) 6C. Results for individual mice are shown, and a horizontal bar represents each median value. [file Image_1.TIF]

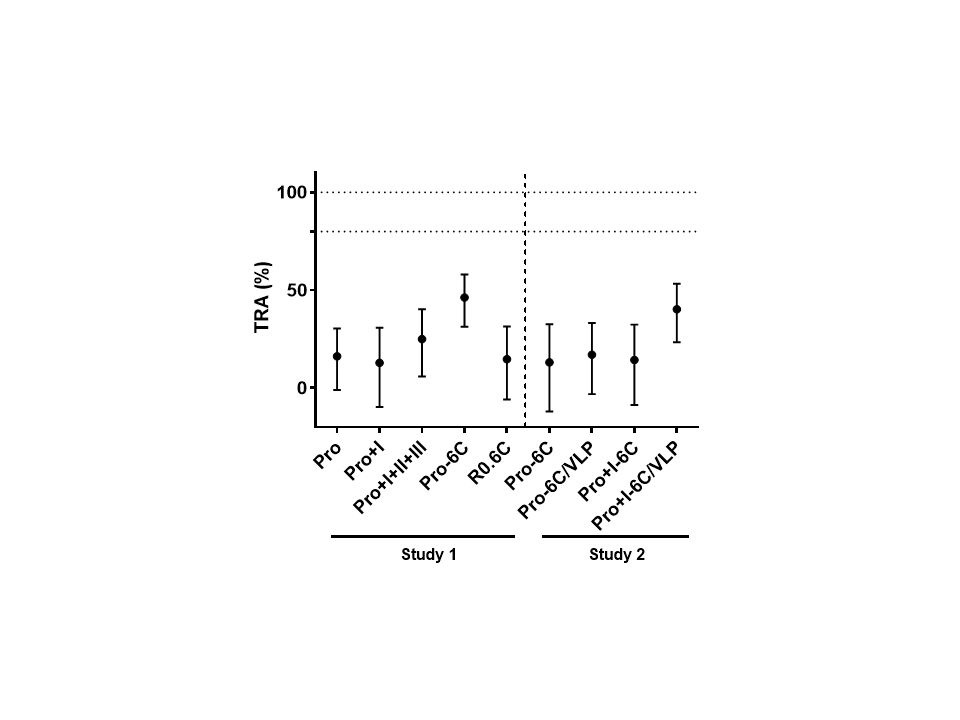

Supplement: Figure S2 — Pre-immune mouse serum contains low intrinsic TRA. Pooled mouse serum from each group was tested at 1/9 dilution in a single SMFA experiment. TRA is the reduction of oocyst numbers compared to a serum control. Error bars represent 95% confidence intervals. TRA values below 50% have large confidence intervals and this can explain the observed variation between different groups. The mean TRA of all groups is 21% (CIs: 5.7–34.1%) demonstrating that mouse serum has low intrinsic TRA. [file Image_2.TIF]
