# Supplementary material for: Pfs230 and Pfs48/45 Fusion Proteins Elicit Strong Transmission-Blocking Antibody Responses Against Plasmodium falciparum
Source: Front Immunol. 2019 Jun 5;10:1256. doi: 10.3389/fimmu.2019.01256 (PMC6560166; doi:10.3389/fimmu.2019.01256)
Supplement: Table S1 — Production and quantification of Pfs230 and Pfs230/Pfs48/45 chimeric protein in L. lactis. [file Data_Sheet_1.PDF]

Table S1. Production and quantification of *Pf*s230 and *Pf*s230/*Pf*s48/45 chimeric protein in *L. lactis*

Constructs	Yield [a] (mg/L)	Monomer [b] (%)	Folding [c] (%)
Pro-6C [d]	15	95.5	100
Pro+I-6C [d]	5	96	100
Pro [e]	30	97	NA
Pro+I [e]	12	96	NA
Pro+I,II,III [e]	3	85	NA

[a] Yield is determined by BCA assay as well as inspection of Coomassie blue stained SDS-PAGE under reducing (10 mM DTT) and or non-reducing conditions using Image Quant TL 8.1 Software (GE Healthcare).
[b] Monomer is given as (amount of total monomer/ amount of total multimer) X 100 % determined by SE-HPLC.
[c] Immune-purified R0.6C used as a reference for calculation of folding.
[d] Immune purification as described (26).
[e] Conventional purification (IMAC followed by IEC)
